# Fatal traumatic brain injuries during 13 years of successive alcohol tax increases in Finland – a nationwide population-based registry study

**DOI:** 10.1038/s41598-019-41913-8

**Published:** 2019-04-01

**Authors:** Jussi P. Posti, Matti Sankinen, Jussi O. T. Sipilä, Jori O. Ruuskanen, Jaakko Rinne, Päivi Rautava, Ville Kytö

**Affiliations:** 1 Division of Clinical Neurosciences, Department of Neurosurgery and Turku Brain Injury Centre, Turku University Hospital and University of Turku, Turku, Finland; 2 Division of Clinical Neurosciences, Department of Neurosurgery, Turku University Hospital and University of Turku, Turku, Finland; 3 Division of Clinical Neurosciences, Department of Neurology, Turku University Hospital and University of Turku, Turku, Finland; 4 Department of Neurology, Siun sote, North Karelia Central Hospital, Joensuu, Finland; 5 Clinical Research Center, Turku University Hospital and University of Turku, Turku, Finland; 6 Heart Center, Turku University Hospital, Turku, Finland; 7 Department of Public Health, University of Turku, Turku, Finland; 8 Research Centre of Applied and Preventive Cardiovascular Medicine, University of Turku, Turku, Finland

## Abstract

We sought to investigate how increases in alcohol taxation and changes in alcohol consumption were associated with the incidence rate of fatal traumatic brain injuries (TBI) in Finland during the years 2004–2016. Nationwide, mandatory cause of death database covering all deaths in Finland was searched for all deaths related to TBIs (ICD-10: S06.X) in persons ≥16 years of age during 2004–2016. Study period included 28,657,870 person-years and 325,514 deaths of which 12,110 were TBI-related. Occurrence rates were standardized to European 2013 standard population. Data for alcohol consumption were obtained from the National Institute for Health and Welfare and for alcohol taxation from Ministry of Finance, Finland. Standardized incidence rate of TBI-related death was 22.0 (95% CI 21.61–22.38) per 100,000 person-years. Overall alcohol consumption decreased on average by 1.2% annually. Concurrently, the overall incidence rate of fatal TBIs decreased by 4.1% annually (by 4.3% in men and 2.4% in women). There was an association between overall alcohol consumption and TBI-related mortality rate (p < 0.001). Tax-rate increases of all beverage types were associated with decreased incidence rate of TBI-related death in men (p < 0.001), in women (p < 0.036) and overall (p < 0.001). In this population-based study, we report that during 13 years of successive alcohol tax increases, overall alcohol consumption has decreased in parallel with a reduction in the incidence rate of fatal TBIs in Finland.

## Introduction

Traumatic brain injury (TBI) is a substantial global health problem and a frequent cause of injury-related death^1,2^. The US Centers for Disease Control and Prevention (CDC) has reported a population-based TBI-related mortality rate of 17.1 per 100,000 person-years in 2010^3^, while in Europe, a pooled mortality rate of 11.7 per 100,000 person-years in 2012 has been reported^2^. Up to half of the patients who sustain TBI are intoxicated with ethanol at the time of injury^4–6^.

Finnish alcohol control has been based on three factors: a strict state-controlled availability of alcohol, a monopoly on alcohol production and trade, and high alcohol beverage prices. Since Finland joined the EU in 1995, the system has partially dissolved. In 2004, the excise tax on alcoholic beverages was reduced markedly and alcoholic beverage prices declined by 33% with the most pronounced relative price reduction in strong alcoholic beverages. This lead to a 10% increase in overall alcohol consumption^7^. In Northern Finland, alcohol-related fatal TBIs increased among middle-aged people in 2006–2007 compared to 1999, while the total number of fatal TBIs remained unchanged^8^. To reduce health hazards, swift increases in alcohol taxes were subsequently undertaken with a focus on strong alcohol taxation. By 2016, overall alcohol taxation had reached almost the same level it had in 2003. The purpose of this study was to investigate possible temporal associations between changes in alcohol taxation and consumption and the rate of fatal TBIs in Finland during the years 2004–2016.

## Materials and Methods

### Study population and alcohol taxation

In Finland, all death certificates must state the cause of death with the ICD-10 code. The death certificates are collected automatically into the Statistics Finland database. We searched this nationwide database covering all deaths in Finland for all underlying causes of death related to acute TBIs (ICD-10 codes S06.0-S06.9) of persons ≥16 years of age during 2004–2016. Data on age- and gender-specific and all-cause mortality of the Finnish population in 2004–2016 were obtained from Statistics Finland. The person-years of each study year were estimated by population at the end of each year. The study was approved by the National Institute for Health and Welfare, Finland (permission no. THL/1484/5.05.00/2017) and Statistics Finland, Finland (TK53-1410-15).

Data on alcohol consumption were obtained from the National Institute for Health and Welfare, Finland, and for alcohol taxation from the Ministry of Finance, Finland. During 2004–2016, the alcohol beverages were divided into taxation group as follows (percentage by volume): beer, cider (≥2.8%), wine (10–14%), medium strong alcohol (21–39%) and strong alcohol (≥40%). The data represents the total consumption of alcoholic beverages in pure alcohol per capita. The data comprises information about documented and undocumented alcohol consumption in Finland. The alcohol sales data are retrieved from the alcohol trade register maintained by The National Supervisory Authority for Wealth and Welfare, Finland. Data on the sales of alcoholic beverages are collected at the municipal level and for the whole country at aggregate level. The data on undocumented consumption are based on Kantar TNS (http://www.tnsglobal.com, 2019) weekly surveys of passenger imports of alcoholic beverages.

### Statistical analysis

Proportions of death and age- and gender-specific incidence rates were calculated. The European 2013 standard population and the direct method were used to standardize the incidence rate of TBI-related deaths. Differences in continuous variables were analyzed with the t-test. The associations between, on the one hand, age, gender, and study year and, on the other hand, the incidence rate and proportion of TBI-related deaths were studied with negative binomial regression modelling. The logarithm of the corresponding population or number of total deaths was used as an offset parameter when modelling incidence rates and the proportionate number of deaths.

Linear regression models were used to analyse the associations between, on the one hand, changes in overall alcohol consumption and changes in alcohol taxation of different alcohol beverages and, on the other hand, the incidence rate of TBI-related deaths. Since data is only available on overall alcohol consumption in the Finnish population, the following associations were studied: (1) changes in overall alcohol consumption with the overall incidence rate of TBI-related deaths, (2) taxation of different beverage types with TBI-related deaths in different age groups by gender. Significance was inferred at p < 0.05. The SAS system version 9.4 (SAS Institute Inc) was used for the statistical analyses.

### Ethics

This is a retrospective registry study and no approval from an ethics committee was required.

## Results

The study period included 28,657,870 person-years and 325,514 deaths. During 2004–2016, TBI-related death certificates were issued in 12,110 cases (68% males). TBI-related deaths occurred mostly (62.3%) in health care facilities (hospitals and health care units), while 21.0% took place in homes or other private apartments, a further 14.8% in other facilities (including nursing homes), 1.0% abroad and 0.9% in social welfare units.

**Figure 1:**
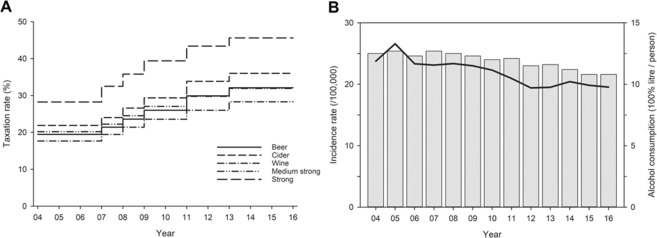
Changes in alcohol taxation of different beverages (**A**), and incidence rate of TBI-related deaths and alcohol consumption (**B**) during the years 2004–2016. In panel the (**B**), the bars represent alcohol comsumption in absolute EtOH and solid line represents incidence rate of TBI-related deaths.

During the study period, alcohol consumption per capita decreased by 1.2% annually (p < 0.001). At the same time, the overall incidence rate of fatal TBIs decreased by 4.1% annually (p < 0.001); the annual decrement rate was 4.3% among males (p = 0.001) and 2.4% among females (p = 0.002). Changes in alcohol taxation, alcohol consumption, and overall incidence rate of fatal TBIs are presented in Fig. 1.

### Incidence rate of fatal traumatic brain injuries and annual trends

The standardized incidence rate of TBI-related deaths was 34.8 (95% CI 34.09–35.47) per 100,000 person-years for males and 12.0 (95% CI 11.59–12.38) per 100,000 person-years for females. The total standardized incidence rate was 22.0 (95% CI 21.61–22.38) per 100,000 person-years. Among both genders, the incidence rate was lowest in the age group 25–34 years with steep increases towards the oldest age group (Table 1).

**Table 1:** Incidence rate (crude) of fatal TBI during 2004–2016 in the Finnish population aged ≥16 years; *per 100,000 person-years.

Age (years)	Men		Women		Total	
	n	Incidence rate (95% CI)*	n	Incidence rate (95% CI)*	n	Incidence rate (95% CI)*
16–24	474	12.08 (11.01–13.21)	127	3.38 (2.82–4.03)	601	7.83 (7.21–8.48)
25–34	438	9.71(8.82–10.66)	69	1.61 (1.26–2.04)	507	5.77 (5.28–6.30)
35–44	572	12.66 (11.64–13.74)	101	2.33 (1.90–2.83)	673	7.60 (7.04–8.20)
45–54	1127	22.96 (21.64–24.34)	206	4.25 (3.69–4.87)	1333	13.66 (12.94–14.42)
55–64	1610	33.50 (31.89–35.18)	313	6.33 (5.65–7.08)	1923	19.73 (18.86–20.64)
65–74	1388	43.24 (40.99–45.57)	476	12.94 (11.80–14.15)	1864	27.06 (25.84–28.31)
75–84	1558	94.45 (89.82–99.26)	1146	44.75 (42.19–47.41)	2704	64.22 (61.82–66.69)
85-	1038	259.95 (244.38–276.26)	1467	135.96 (129.09–143.10)	2505	169.45 (162.88–176.22)
Total Crude	8205	29.38 (28.75–30.02)	3905	13.25 (12.83–13.67)	12110	21.10 (20.72–21.48)
Total Standardized	—	34.80 (95% CI 34.09–35.47)	—	12.00 (95% CI 11.59–12.38)	—	22.00 (95% CI 21.61–22.38)

**Figure 2:**
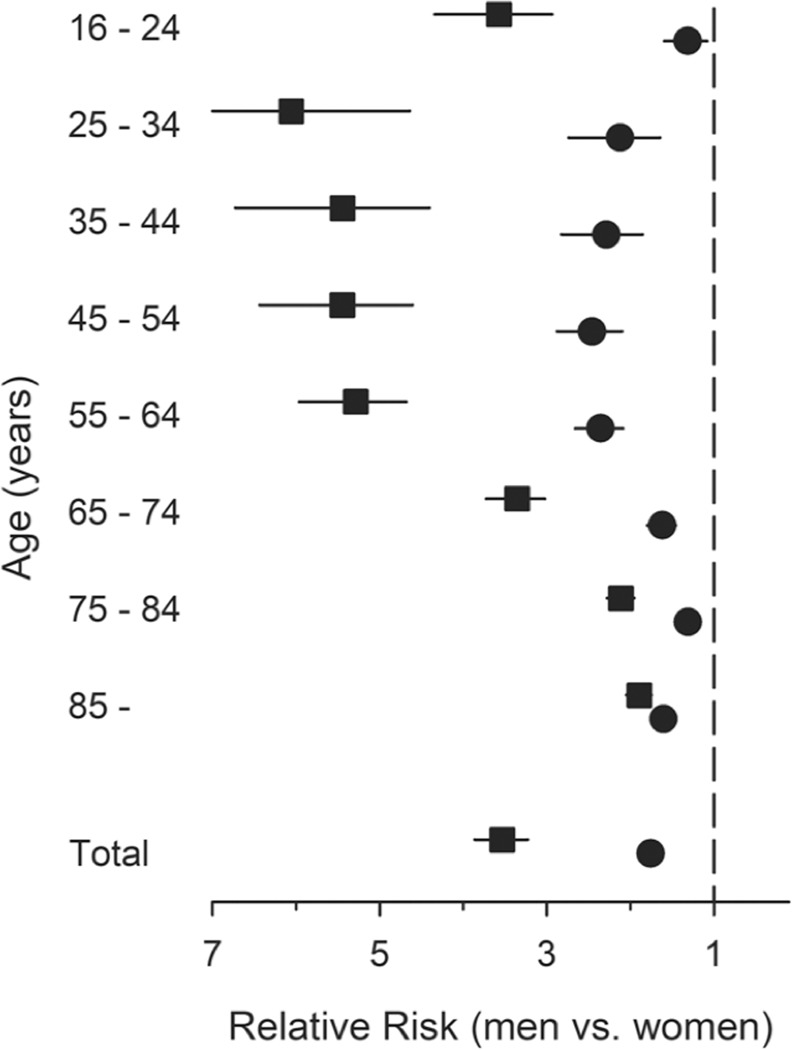
Gender differences in relative risk for TBI-related death by age. Squares represent TBI incidence rate in general population and circles represent death to be TBI-related of all deceased. Results are adjusted for study year and for age (total relative risk). Error bars represent 95% confidence intervals.

Males had a 3.5-fold (95% CI 3.22–3.86; p < 0.001) relative risk of TBI compared to women. This gender-based risk difference was age-dependent (interaction p < 0.001) and was greatest in the age group 25–34 years (IRR 6.06) and lowest (IRR 1.89) in the oldest age group (≥85 years) (Fig. 2).

During the study period, the incidence rate of fatal TBIs decreased in males aged 16–74 years (p < 0.001), but increased in males aged ≥85 years (p ≤ 0.025) (Table 2). The annual incidence decrement was highest (−10.3%) in the youngest male age group. Among females, the incidence rate of fatal TBIs decreased in the age groups 16–54 years (p ≤ 0.010) and 65–74 years (p ≤ 0.003), but increased in females aged ≥85 years (p < 0.001). The total incidence rate of fatal TBIs in males and females decreased when analysed separately (p < 0.001 and p < 0.002, respectively) and together (p < 0.001). The annual incidence trend of fatal TBIs varied between genders (interaction p = 0.029) (Table 2). The total incidence rate of fatal TBIs remained above 23.00 per 100,000 person-years until 2009 and started to decline thereafter (Table 3 and Fig. 1). Only in individuals aged ≥75 years, the incidence rate of fatal TBIs increased (Table 3).

**Table 2:** Estimated annual trends of the fatal TBI incidence rate during 2004–2016 in the Finnish population aged ≥16 years. The annual trend varied between the genders (interaction p = 0.029). *sex-adjusted; **age-adjusted.

Age (years)	Men		Women		Total*	
	Annual change % (95% CI)	p value	Annual change % (95% CI)	p value	Annual change % (95% CI)	p value
16–24	−10.34 (−12.58–−8.04)	<0.0001	−7.21 (−12.33–−1.8)	0.0097	−9.65 (−11.65–−7.6)	<0.0001
25–34	−6.21 (−8.56–−3.79)	<0.0001	−14.90 (−22.36–−6.72)	0.0006	−7.52 (−10.14–−4.83)	<0.0001
35–44	−7.97 (− 10.02–−5.88)	<0.0001	−11.24 (−16.03–−6.18)	<0.0001	−8.46 (−6.53–−10.34)	<0.0001
45–54	−7.06 (−9.10–−4.98)	<0.0001	−10.13 (−13.52–−6.60)	<0.0001	−7.75 (−9.50–−5.96)	<0.0001
55–64	−4.14 (−5.41–−2.86)	<0.0001	−2.35 (−5.24–0.64)	0.1222	−3.85 (−5.02–−2.67)	<0.0001
65–74	−4.01 (−5.39–−2.61)	<0.0001	−3.54 (−5.81–−1.22)	0.0029	−3.89 (−5.05–−2.72)	<0.0001
75–84	−0.48 (−1.79–0.86)	0.4818	1.03 (−0.52–2.60)	0.1935	0.16 (−0.84–1.18)	0.7516
85-	2.46 (0.31–4.66)	0.0246	3.41 (1.96–4.87)	<0.0001	2.96 (1.85–4.07)	<0.0001
Overall**	−4.29 (−5.21–−3.35)	<0.0001	−2.43 (−3.94–−0.90)	0.0019	−4.08 (−5.19–−2.96)	<0.0001

**Table 3:** Annual breakdown of the incidence rate of fatal TBIs during 2004–2016 in the Finnish population aged ≥16 years; *per 100,000 person-years.

Year	Adjusted total incidence rate*	Incidence rate by age groups*							
		16–24 years	25–34 years	35–44 years	45–54 years	55–64 years	65–74 years	75–84 years	85- years
2004	23.75	13.30	6.75	12.89	17.43	22.03	32.85	60.20	123.98
2005	26.59	10.03	8.75	9.65	20.72	26.72	36.59	66.04	167.06
2006	23.33	10.83	7.27	9.83	19.02	23.09	32.26	60.01	123.74
2007	23.11	11.67	8.96	9.17	16.68	21.10	28.75	63.00	138.36
2008	23.37	11.30	7.71	9.85	16.45	21.42	28.13	62.14	155.11
2009	23.00	7.59	5.27	8.21	16.13	20.51	30.62	62.50	174.81
2010	22.30	6.38	6.42	7.53	13.70	18.32	28.49	67.52	175.19
2011	20.95	7.18	4.81	5.61	12.18	19.90	22.21	67.77	172.12
2012	19.40	5.67	3.94	5.63	7.25	18.47	23.00	62.86	175.59
2013	19.51	5.03	5.36	5.18	9.28	19.06	24.82	61.73	158.05
2014	20.42	3.90	2.30	5.31	8.98	16.62	24.70	72.54	193.97
2015	19.83	3.95	3.72	3.92	8.42	15.95	25.06	63.56	203.24
2016	19.53	4.87	4.40	4.92	10.24	13.84	20.76	64.15	197.37

### Proportions and annual trends of deaths caused by traumatic brain injuries

The proportion of deaths related to TBI was 1.9%. For both genders, the proportion of fatal TBIs was highest in the age group 16–24 years. Overall, males had a 75% higher relative risk (RR 1.75; 95% CI 1.64–1.86; p < 0.0001) of TBI-related death. The gender difference was age-dependent (interaction p < 0.001) with the highest difference between 45–54 years and lowest between 75–84 years (Fig. 2).

The proportion of TBI-related deaths was halved in both genders after age of 24 years, after which the proportions continued to decline towards the oldest age group during the study period (Table 4).

**Table 4:** Proportion (%) of deaths caused by TBI during 2004–2016 in the Finnish population aged ≥16 years.

Age (years)	Men		Women		Total	
	n	% (95% CI)	n	% (95% CI)	n	% (95% CI)
16–24	474	15.68 (14.30–17.15)	127	11.80 (9.84–14.04)	601	14.66 (13.51–15.88)
25–34	438	9.11 (8.28–10.01)	69	4.28 (3.33–5.41)	507	7.90 (7.22–8.62)
35–44	572	6.68 (6.14–7.25)	101	2.93 (2.39–3.56)	673	5.60 (5.19–6.04)
45–54	1127	4.97 (4.68–5.27)	206	2.02 (1.76–2.32)	1333	4.05 (3.84–4.28)
55–64	1610	3.19 (3.04–3.35)	313	1.35 (1.21–1.51)	1923	2.61 (2.50–2.73)
65–74	1388	1.95 (1.85–2.05)	476	1.20 (1.10–1.32)	1864	1.68 (1.61–1.76)
75–84	1558	1.58 (1.51–1.66)	1146	1.20 (1.13–1.27)	2704	1.40 (1.34–1.45)
85-	1038	1.56 (1.47–1.66)	1467	0.97 (0.92–1.02)	2505	1.15 (1.10–1.19)
Total	8205	2.52 (2.47–2.58)	3905	1.20 (1.16–1.24)	12110	1.86 (1.83–1.89)

The annual trend varied between the genders (interaction p = 0.0051); *sex-adjusted; **age-adjusted.

The proportion of deaths that were related to TBI decreased in males aged ≤74 years (p ≤ 0.020), whereas in older males the proportion increased (p ≤ 0.027). The overall proportion decreased in males (annual change −1.6%, p < 0.001). The proportion of deaths related to TBI decreased in females aged 25–54 years (p ≤ 0.011) and increased in females aged ≥75 years (p ≤ 0.001). There was an annual overall decrement of 1.4% (Table 5).

**Table 5:** Estimated annual trends of the proportion of deaths caused by TBI during 2004–2016 in the Finnish population aged ≥16 years.

Age (years)	Men		Women		Total*	
	Annual change % (95% CI)		Annual change % (95% CI)	p value	Annual change % (95% CI)	p value
16–24	−6.76 (−9.15–−4.30)	<0.0001	−4.68 (−9.43–0.32)	0.0659	−6.29 (−8.41–−4.12)	<0.0001
25–34	−4.95 (−7.40–−2.44)	0.0001	−14.21 (−21.80–−5.88)	0.0012	−6.15 (−8.53–−3.71)	<0.0001
35–44	−3.94 (−6.09–−1.73)	0.0005	−7.03 (−12.10–−1.68)	0.0107	−4.39 (−6.39–−2.35)	<0.0001
45–54	−2.99 (−4.81–−1.13)	0.0017	−6.92 (−10.45–−3.25)	0.0003	−3.73 (−5.39–−2.04)	<0.0001
55–64	−2.40 (−3.71–−1.07)	0.0004	−1.21 (−4.17–1.84)	0.4310	−2.20 (−3.49–−0.94)	0.0007
65–74	−1.72 (−3.13–−0.28)	0.0196	−2.07 (−4.36–0.27)	0.0826	−1.81 (−2.99–−0.61)	0.0033
75–84	1.51 (0.17–2.88)	0.0274	2.72 (1.15–4.32)	0.0007	2.03 (1.00–3.07)	<0.0001
85-	3.30 (1.01–5.65)	0.0045	3.83 (2.38–5.29)	<0.0001	3.52 (2.41–4.64)	<0.0001
Overall**	−1.64 (−2.43–−0.86)	<0.0001	−0.16 (−1.53–1.23)	0.8171	−1.39 (−2.19–−0.59)	0.0007

### Changes in alcohol consumption, alcohol taxation, and incidence rate of fatal traumatic brain injuries

There was an association between the overall reduction in alcohol consumption and the reduction in the TBI-related mortality rate (p < 0.001). Increases in the taxation of all beverage types were associated with a reduced incidence rate of TBI-related death in males (p < 0.001), females (p < 0.036), and overall (p < 0.001).

The incidence rate of fatal TBIs decreased among males aged 16–74 years and among females aged 16–54 and 65–74 years. In all these age groups, there was an association between the changes in alcohol taxation of the different beverage types and the incidence rate of fatal TBIs (Table 6).

**Table 6:** Associations between changes in incidence rate of TBI-related deaths by gender, changes in overall alcohol consumption, and changes in alcohol taxation of different alcohol beverages during 2004–2016.

		Overall alcohol consumption		Alcohol taxation									
				Strong alcohol		Medium strong alcohol		Wine		Cider		Beer	
		Parameter estimate	p value	Parameter estimate	p value	Parameter estimate	p value	Parameter estimate	p value	Parameter estimate	p value	Parameter estimate	p value
Total	Total	2.75090	0.0002	−0.26731	<0.0001	−0.39942	<0.0001	−0.44205	<0.0001	−0.33057	<0.0001	−0.36961	<0.0001
	Men	5.61930	<0.0001	−0.50965	0.0002	−0.77064	0.0001	−0.85757	0.0001	−0.64312	<0.0001	−0.72058	<0.0001
	Women	0.44999	0.0972	−0.05571	0.0184	−0.07838	0.0297	−0.08415	0.0376	−0.06356	0.0326	−0.07011	0.0363
Age 16–24	Total			−0.42260	<0.0001	−0.63372	<0.0001	−0.70342	<0.0001	−0.51816	<0.0001	−0.58053	<0.0001
	Men			−0.70381	<0.0001	−1.05087	<0.0001	−1.16304	<0.0001	−0.86141	<0.0001	−0.96351	<0.0001
	Women			−0.12856	0.0411	−0.19760	0.0351	−0.22293	0.0320	−0.15932	0.0396	−0.18017	0.0376
Age 25–34	Total			−0.24625	<0.0001	−0.37089	<0.0001	−0.41324	<0.0001	−0.30366	<0.0001	−0.34091	<0.0001
	Men			−0.35697	<0.0001	−0.53890	<0.0001	−0.60143	<0.0001	−0.44104	<0.0001	−0.49565	<0.0001
	Women			−0.13133	0.0022	−0.19639	0.0022	−0.21774	0.0024	−0.16095	0.0023	−0.18015	0.0024
Age 35–44	Total			−0.33546	<0.0001	−0.50298	<0.0001	−0.55802	<0.0001	−0.41186	<0.0001	−0.46131	<0.0001
	Men			−0.53172	<0.0001	−0.79885	<0.0001	−0.88796	<0.0001	−0.65529	<0.0001	−0.73477	<0.0001
	Women			−0.13606	0.0016	−0.20232	0.0019	−0.22270	0.0022	−0.16444	0.0022	−0.18336	0.0025
Age 45–54	Total			−0.57664	<0.0001	−0.86188	<0.0001	−0.95412	<0.0001	−0.71346	<0.0001	−0.79784	<0.0001
	Men			−0.90737	<0.0001	−1.35620	<0.0001	−1.50026	<0.0001	−1.12583	<0.0001	−1.25827	<0.0001
	Women			−0.24483	<0.0001	−0.36605	<0.0001	−0.40637	<0.0001	−0.29976	<0.0001	−0.33596	<0.0001
Age 55–64	Total			−0.39921	<0.0001	−0.60410	<0.0001	−0.67559	<0.0001	−0.49251	<0.0001	−0.55442	<0.0001
	Men			−0.73440	0.0001	−1.11305	<0.0001	−1.24479	<0.0001	−0.90914	0.0001	−1.02295	0.0001
	Women			−0.06570	0.2384	−0.09795	0.2410	−0.10964	0.2380	−0.07823	0.2555	−0.08857	0.2508
Age 65–74	Total			−0.53667	0.0003	−0.79634	0.0004	−0.88063	0.0005	−0.64988	0.0005	−0.72753	0.0005
	Men			−0.93248	0.0014	−1.38975	0.0016	−1.54018	0.0017	−1.12658	0.0020	−1.26286	0.0020
	Women			−0.25130	0.0067	−0.36778	0.0087	−0.40404	0.0100	−0.30681	0.0074	−0.34210	0.0079
Age 75–84	Total			0.21090	0.1317	0.32211	0.1236	0.36071	0.1209	0.24864	0.1510	0.28089	0.1478
	Men			−0.15261	0.6227	−0.25186	0.5874	−0.28782	0.5772	−0.24654	0.5169	−0.27653	0.5169
	Women			0.24861	0.0782	0.39846	0.0566	0.45256	0.0507	0.32810	0.0561	0.37125	0.0535
Age 85-	Total			2.93458	0.0004	3.55944	0.0006	4.94075	0.0003	3.55944	0.0006	4.01205	0.0005
	Men			3.85686	0.0252	5.63691	0.0301	6.22331	0.0318	4.35531	0.0446	4.89086	0.0443
	Women			2.21481	0.0007	3.38636	0.0004	3.81700	0.0003	2.78603	0.0004	3.14791	0.0003

## Discussion

We investigated how increases in alcohol taxation and alterations in overall alcohol consumption were associated with the rate of fatal TBIs in Finland in the years 2004–2016. During these years, alcohol taxation was successively increased, while the compound annual rate of overall alcohol consumption fell by 1.2%. Concurrently, the overall incidence rate of fatal TBIs decreased by 4.1% annually, 4.3% in males and 2.4% in females. Tax-rate increases of all beverage types were associated with a reduction of the incidence rate of overall deaths and TBI-related deaths in both genders. Reduced overall alcohol consumption was associated with reduced TBI-related mortality.

Alcohol intoxication increases the susceptibility to injuries and accidents. Nearly half of the patients who sustain TBI are under the influence of alcohol at the time of injury^4–6^, and, clearly, alcohol intake may be considered to be a major health problem and alcohol intoxication is a risk factor for TBI-related mortality and injury-related complications^9^. Alcohol abuse occurs among 44% to 66% of the patients with TBI^10^. Moreover, a recent study reported that there was a systemic bias per sex and age for alcohol screening in a relatively large American dataset: patients with mild or severe injuries, midrange state of consciousness, younger patients, and women were less likely to be screened^11^. Hospital records may therefore underestimate the prevalence of alcohol intoxication among the patients with acute TBI.

Despite this overwhelming data on the association between alcohol consumption and TBI, the associations between restricted alcohol availability and fatal TBIs are poorly known. In Northern Finland, after the substantial reduction of alcohol taxes in 2004, alcohol-related fatal TBIs increased among middle-aged people but the total number of fatal TBIs did not^8^. On a global scale, road injuries are the second most common alcohol-attributable cause of death among individuals aged 15–49 years^12^. In the United States, stricter alcohol policies, including increased alcohol taxes and restriction of alcohol beverage availability, have resulted in reduced odds of alcohol-related car crash deaths^13^. In the case of Finland, the strict state-controlled availability of alcohol, in addition to successive increases in alcohol taxation and the concomitant reduction in alcohol consumption, are the most likely reason for the decreased incidence rate of fatal TBIs during 2004–2016.

The total incidence rate of TBI-related deaths reported in this paper is 22.0 per 100,000 person-years. This figure is slightly higher than the 21.8 per 100,000 upper limits of the variation reported for the European countries^2^. It is also higher than the rates of 17.8 and 18.3 previously reported for Finland^2,14^. However, these studies also included pediatric patients among whom the incidence rate is very low, which confounds direct comparisons with our results. TBI-related mortality was reportedly three times higher in Finland than in the other Nordic countries in 1987–2001 (9.0 in Sweden and 8.7 in Denmark)^15^. A partial explanation for this finding may be the high autopsy rate in Finland, which was almost two times higher than in other Nordic countries at the time of the above studies since the early 90’s (World Health Organization, European Health Information Gateway, https://gateway.euro.who.int/en/, 2019).

In the present study, the incidence rate of TBI-related deaths was lowest in both genders in the age group 25–34 years. The incidence rate rose steeply toward the oldest age group, which is in accordance with American^16^ Canadian^17^, and Dutch results^18^. According to a previous study from Finland, the TBI-related mortality risk by age for males is highest in age groups 40–49 and 50–59 years; in women, the mortality risk increases after age 70^14^. In the context of these studies and current results, tightening alcohol taxation shows no temporal association with the incidence rate of TBI in elderly.

We observed that males have a 3.5-fold overall relative risk of TBI compared to women. The extent of this gender-based risk difference is age-dependent with the highest difference in age groups 25–34 years and the lowest difference in the age group ≥85 years. The standardized incidence rate of fatal TBIs was 34.8 per 100,000 person-years among males and 12.0 among females. In a dataset covering 25 European countries, TBI-related mortality followed a similar gender and age pattern^2^.

Studies on the epidemiology of TBI-related deaths by geography are rare. In this study we found that the proportion of deaths related to TBI in Finland was 1.9% overall, for males 2.5%, and for females 1.2%. The proportion of fatal TBIs was highest in the youngest age group in both genders, exceeding 11%. The proportion of TBI-related deaths was approximately halved in both genders after age 24 years, after which the proportions continued to decline towards the oldest age group.

The alcohol taxes were not incereased before 2007. However, the timeframe for this study was chosen to comprise the years before the alcohol tax increases in order to detect the impact of the tax reduction made at the beginning of 2004. Hence, we aggregated the 13-year period in the trend analysis. Some variation in the incidence rate of fatal TBIs and alcohol consumption rates during the years 2004–2007 can be observed, but the decline in the incidence rate of fatal TBIs can be seen after 2009. While the decline in the incidence rate is clear in the younger age groups, in individuals aged ≥75 years, the incidence rate of fatal TBIs increased during the years 2004–2016.

The current results in terms of TBI-related deaths can be regarded as being population-based, since a death certificate which states the cause of death and collects all cases of death in Finland is obligatory. There are, however, some methodological limitations in this study. Due to the retrospective register study setting and the administrative nature of data there is naturally some uncertainty. The data from the nationwide cause of death database covering all deaths in Finland is only available from the year 2004, which prevents the estimation of the effect of the tax decreases on incidence rate of fatal TBIs compared to the circumstances before the alcohol tax reduction. Alcohol involvement in the TBI-related cases of death has not been examined. The data on alcohol consumption is available only for overall usage, and hence we have conducted association analyses mainly between changes in alcohol taxation and changes in fatal TBIs. This is a shortcoming, since it does not identify a causal association. We do not claim that the decreased incidence rate of fatal TBIs is mainly related to a successive tightening of alcohol taxes and reduced overall consumption. However, these phenomena have occurred simultaneously.

## Conclusions

During 13 years of successive alcohol tax increases, overall alcohol consumption decreased annually by 1.2%, and concurrently the overall incidence rate of fatal TBIs decreased by 4.1% annually (4.3% in males and 2.4% in females). There was a temporal association between the decrease in overall alcohol consumption and TBI-related mortality. Tax-rate increases of all beverage types were associated with a decreased incidence rate of TBI-related deaths overall and for both genders separately.

Stricter alcohol policies, as introduced in Finland, including policies that do not specifically target excessive binge drinking, may reduce alcohol-related TBI fatalities. These findings may apply to other societies, as well.

### Ethical approval and informed consent

This is a retrospective registry study and no approval from an ethics committee was required.

## Data Availability

Due to national data protection legislation, the register data used in this study cannot be shared without applying for permission to use the data with a specific study protocol and scientifically justified study questions.
